# Biogenesis of Prism-Like Silver Oxide Nanoparticles Using Nappa Cabbage Extract and Their *p*-Nitrophenol Sensing Activity

**DOI:** 10.3390/molecules25102298

**Published:** 2020-05-13

**Authors:** Jomaris Banua, Jeong In Han

**Affiliations:** Department of Chemical and Biochemical Engineering, Dongguk University—Seoul, Seoul 04620, Korea; jombanua19@gmail.com

**Keywords:** silver oxide nanoparticles, biogenesis, nappa cabbage, *p*-nitrophenol sensor

## Abstract

The present study aimed to explore the eco-friendly synthesis of prism-like silver oxide nanoparticles (Ag_2_ONPs) from nappa cabbage extract and its *p*-nitrophenol sensing activity. The prepared Ag_2_ONPs were characterized by X-ray diffraction (XRD), field-emission scanning spectroscopy (FESEM), energy-dispersive spectroscopy (EDS), transmission electron microscopy (TEM), and ultraviolet (UV)–visible light spectral analysis (UV–Vis). *p*-Nitrophenol sensing properties of the prepared nanoparticles were also determined using a simple I–V method. The results showed that the as-prepared Ag_2_ONPs have a face-centered cubic (fcc) crystalline nature and a prism-like morphology with particle size in the range 21.61–92.26 nm. The result also showed a high intensity of the (111) facet, making the Ag_2_ONP–carbon black/nickel foam electrode (Ag_2_ONP–C/NFE) exhibit a high-performance response to *p*-nitrophenol spanning a wide range of concentrations from 1.0 mM to 0.1 pM and a response time of around 5 s, indicating a high potential for water treatment applications.

## 1. Introduction

Over the past couple of decades, industrialization resulted in increasing quantities of organic pollutants in waterbodies, imposing detrimental effects to the ecosystem [1,2]. Among these is the environmental pollution caused by the untreated discharge of dyes from manufacturing industries which can impede the photosynthesis of aquatic organisms [3,4,5]. Globally, water pollution is one among the most crucial issues owing to the discharge of toxic effluents posing greater threats to human health [6]. Nitrophenols are extensively used in several petrochemical syntheses, including plastics, dyes, rubber, pulp, paints, and pesticides [7,8]. In particular, *p*-nitrophenol (*p*-NP, 4-nitrophenol, PNP) can cause an intensive effect on methemoglobin formation, triggering skin and eye irritation, anemia, liver and kidney damage, and systematic poisoning [9]. Furthermore, it was found that, in the bodies of water, *p*-NP concentration and exposure time are directly proportional to the mortality rate of fishes [10]. Because of its harmful impacts on the growth and metabolism of plants, animals, and human beings, it is listed by the United States (US) Environmental Protection Agency (EPA) as one of the priority pollutants [11,12]. For years, several methods of minimizing nitrophenol pollution in water were developed, including adsorption [13], photo-degradation [14], chemical oxidation [15], etc. However, for a successful elimination method of pollutants, all these methods must always be accompanied by a detection and measurement method. Previous reports on the detection of *p*-NP focused on gas chromatography–mass spectroscopy [16], high-performance liquid chromatography [17], ultraviolet–visible light (UV–Vis) spectrophotometry [18], liquid chromatography [19], fluorescence detection [20], and capillary electrophoresis [21]. Because of the long duration of analysis, the complexity of sample preparation, and the high cost of the machines, the quick and cost-efficient quantification of *p*-NP is an obstacle to manufacturing plants. Therefore, it is necessary to develop an accurate, selective, fast, easy, cost-effective, and portable analytical measurement of the concentration of *p*-NP without causing extra pollution. With this, an electrochemical sensing method of *p*-NP pollutant gained huge attention in the detection of *p*-NP because of the advantages of fast response, simple operation, inexpensive instrument, low analysis cost, and time saving, along with real-time detection in situ conditions [22,23]. The determining factor for the success of these electrochemical sensors is the sensing electrode used. The development of novel electrode materials for the modification of electrodes is the most important factor to enhance their electrochemical performance, which is found to be significantly dependent on the microstructures of the electrode materials. Thus, a variety of functional materials, particularly metal materials and carbon materials, were designed and synthesized for constructing a novel electrode system [24]. 

A chemical sensor is a sensor that produces an electric signal directly proportional to the concentration of chemical analytes. Metal oxide nanostructures have unique properties, such as good electrical conductivity, magnetic properties, optical properties, and outstanding catalytic activity. Recently, transition metal oxide-doped semiconductor materials received significant interest in developing their physico-chemical behaviors and extending their efficient chemical sensor applications [25,26,27]. In particular, the silver–oxygen system (Ag–O) gained extensive attraction by researchers due to its novel applications in gas sensors, high-density optical storage devices, photovoltaic cells, antibacterial coatings, photo diodes, and chemical sensors [28,29]. This system exists in different defined compounds, namely, Ag_2_O, AgO, Ag_3_O_4_, Ag_4_O_3_, Ag_2_O_3_, and Ag_4_O_4_. However, the most thermodynamically stable among these compounds is Ag_2_O which possesses a simple cubic structure at room temperature [30]. The performance of Ag_2_O-based sensors highly depends on the specific surface area of the particle which can be tuned by controlling its microstructure in terms of morphology and porosity at the nanolevel. Furthermore, Ag_2_O is already utilized for its bacterio-static properties [31], giving the material wide potential applications such as wastewater treatment, potable water monitoring, and medical devices. With this, scientists reported several methods to synthesize silver oxide nanoparticles including a sonochemical method [32], galvanosynthesis [33], green synthesis [34], etc. Due to our aim of creating an effective *p*-nitrophenol sensor without causing extra pollution in the fabrication process, we chose green synthesis. Using plant extract to reduce metal salts gives the advantage of producing stabilized nanoparticles without using any stabilizing agent [35].

In this paper, we propose a facile biological synthesis of prism-like silver oxide nanoparticles (Ag_2_ONPs) used as sensing material for electrode modification for the detection of *p*-nitrophenol. The as-prepared Ag_2_ONPs were synthesized by reducing AgNO_3_ precursor with nappa cabbage extract. Nappa cabbage, *Brassica rapa* L. subsp. *pekinensis*, is a commonly used vegetable in Korea [36]. Studies reported that nappa cabbage is rich in natural phenolic compounds such as phenols, phenolic acid, hydroxycinnamic acid derivatives, and flavonoids [37]. In connection to this, the ability of plant extracts to act as reducing and capping agents in the plant-mediated synthesis of metals and metal oxides is due to the presence of these complex compounds possessed by plants [38,39,40]. Most of the previous reports incorporate a boiling step to get an extract from the specific plant used [41]. Moreover, it was found that phytochemicals found in plants and vegetables are susceptible to destruction upon boiling [42]. Since the key factors in the biological reduction process are these natural compounds, protecting these compounds from destruction is very important. Thus, we opted for a boiling step during extraction. With this, we decided to choose a plant that has high liquid content to easily get an extract without boiling in the extraction process. One of the plants that has high enough liquid content and is a highly used vegetable in Korea and other countries is the nappa cabbage, which is also one of the dominating sources of waste in traditional markets [43]. To the best of our knowledge the biosynthesis of silver oxide using nappa cabbage extract was never previously reported.

## 2. Results

### 2.1. X-ray Diffraction (XRD) Analysis of Nappa Cabbage-Mediated Biosynthesis of Prism-Like Silver Oxide Nanoparticles (Ag_2_ONPs)

**Figure 1:**
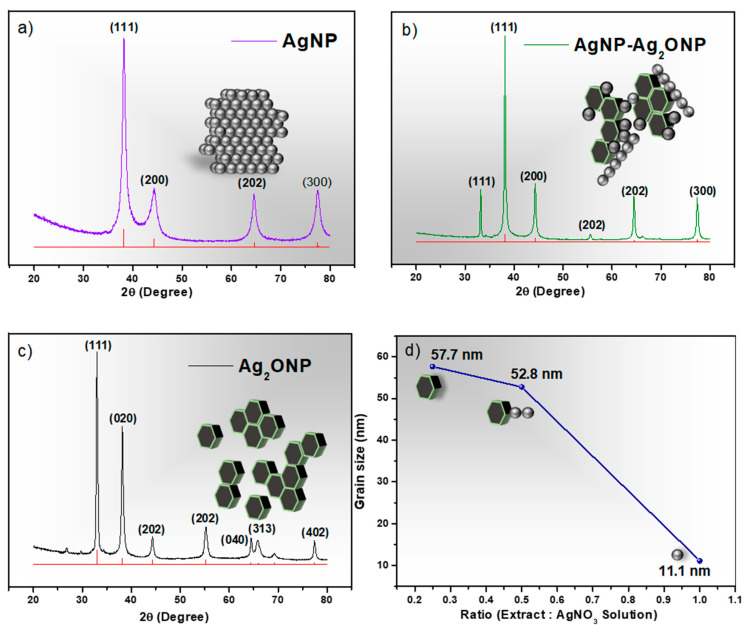
X-ray diffraction (XRD) patterns of synthesized nanoparticles at different extract ratios: (**a**) AgNP at 1:1 ratio; (**b**) AgNP–Ag_2_ONP at 1:2 ratio; (**c**) Ag_2_ONP at 1:4 ratio; (**d**) grain size vs. extract ratio of synthesized nanoparticles.

To synthesize the stable prism-like silver oxide nanoparticles, a facile biological synthesis method was employed. The as-prepared Ag_2_ONP was found to exhibit a high sensitivity to the detection of low concentration of *p*-NP. Furthermore, the resulting nanoparticle showed a dependency on the amount of extract used. In order to comprehend the significance of extract concentration in the biological reduction process, different amounts of extract were used (40 mL, 20 mL, and 10 mL) with a fixed amount of AgNO_3_ solution. The XRD patterns of the synthesized nanoparticles at varying extract concentration shown in Figure 1 confirm the effect of the amount of extract in the biological synthesis of Ag_2_ONPs.

Figure 1a shows the XRD pattern of the sample having an extract-to-AgNO_3_-solution ratio of 1:1. It shows four peaks at 2θ = 38.18°, 43.34°, 64.48°, and 77.46° corresponding to the (111), (200), (202), and (300) planes of Ag XRD pattern with JCPDS no. 96-110-0137, having no extra peak detected, which confirmed the formation of pure Ag nanoparticles using the ratio mentioned. This suggests that, using a 1:1 volume ratio, all the silver atoms in the solution were completely biologically reduced to their elemental form by the extract. When the volume of extract was halved giving a ratio of 1:2 extract to AgNO_3_ solution, the diffraction peaks of the resulting nanoparticles were observed again, as shown in Figure 1b. The XRD pattern detected two unknown peaks with respect to the same Ag XRD pattern basis (JCPDS no. 96-110-0137). The 1:2 ratio shows the same four peaks corresponding to (111), (200), (202), and (300) planes which were assigned to the lattice representative of Ag. However, two new prominent peaks were detected at 2θ = 33.17° and 55.54° corresponding to (111) and (202), which belong to Ag_2_O (JCPDS no. 96-101-0487). This suggests that reducing the amount of extract in the reactional mixture allows the formation of silver oxide which is the material of interest in this research. Finally, further reduction of the extract volume to 10 mL giving a ratio of 1:4 extract to AgNO_3_ solution gave the XRD pattern shown in Figure 1c. The 1:4 ratio had seven prominent peaks at 2θ = 33.18°, 38.16°, 44.32°, 55.54°, 64.44°, 66.26°, and 77.40° conforming to (111), (020), (202), (202), (040), (313), and (402) planes of Ag_2_O. Furthermore, there was a peak at 2θ = 26.88° corresponding to the (101) plane. The presence of this peak could be incorporated into the plant extract which contains organic compounds, and it is responsible for the reduction of silver ions and stabilization of resultant nanoparticles [18]. The XRD results show that the transition of the resulting nanoparticles from Ag to Ag_2_O is caused by the amount of extract used in the mixture.

### 2.2. Field-Emission Scanning Spectroscopy (FESEM) and Energy-Dispersive Spectroscopy (EDS) Analyses of Nappa Cabbage-Mediated Biosynthesis of Prism-Like Silver Oxide Nanoparticles (Ag_2_ONPs)

**Figure 2:**
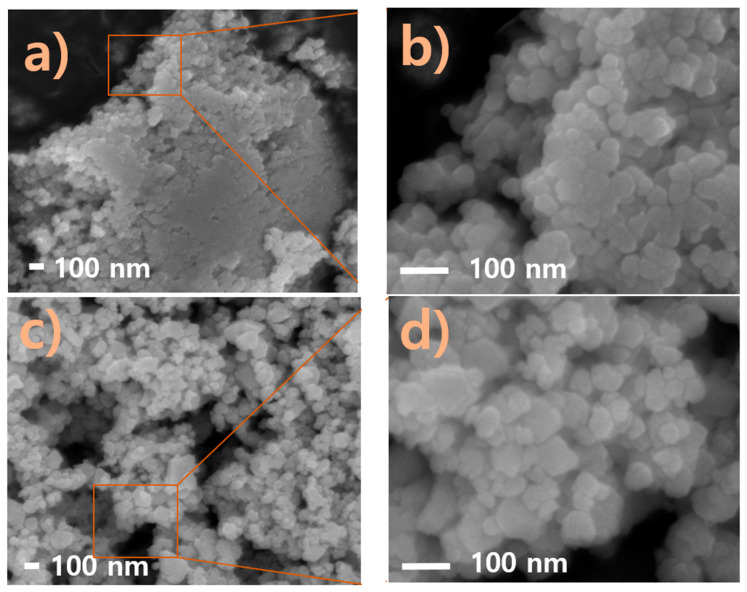

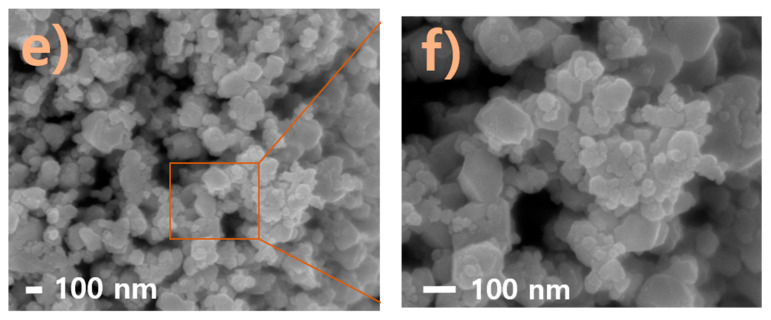
Field-emission scanning electron microscope (FESEM) images of synthesized nanoparticles at different extract ratios: (**a**) AgNP at 1:1 ratio; (**b**) magnified image of 1:1; (**c**) AgNP–Ag_2_ONP at 1:2 ratio; (**d**) magnified image of 1:2; (**e**) Ag_2_ONP at 1:4 ratio; (**f**) magnified image of 1:4.

**Figure 3:**
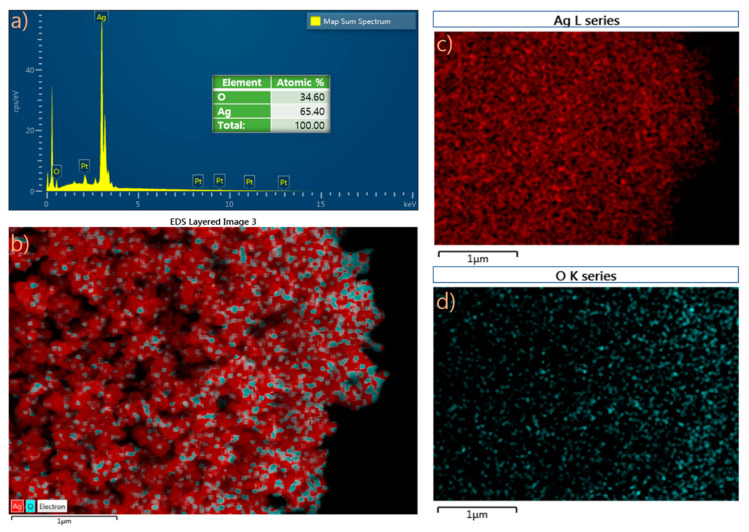
Energy-dispersive spectroscopy (EDS) elemental mapping of 1:4 ratio: (**a**) EDS spectrum and EDS element mapping images of combined O and Ag, (**b**) O, (**c**) and (**d**) Ag.

FESEM (Figure 2a) clearly shows the presence of synthesized silver nanoparticles. The nanoparticles were oval and spherical in shape. Most of the nanoparticles were aggregated, and a few individual particles were also observed. In addition, the 1:2 ratio (Figure 2b) showed the presence of a few particles with prism-like nanoparticles. Finally, shown in Figure 2c is the FESEM image of the prism-like silver oxide nanoparticles with an average grain size of 57 nm and particle size ranging from 21.61–92.26 nm. Additionally, to comprehend the atomic composition of the prepared nanoparticles, the EDS elemental mapping of the 1:4 ratio was investigated as shown in Figure 3.

### 2.3. Transmission Electron Microscopy (TEM) Analysis of Nappa Cabbage-Mediated Biosynthesis of Prism-Like Silver Oxide Nanoparticles (Ag_2_ONPs)

**Figure 4:**
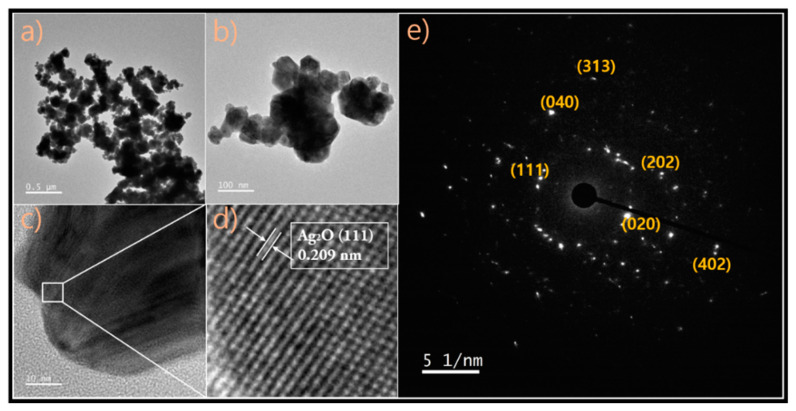
TEM images of synthesized Ag_2_ONP: (**a**) low-magnification TEM image of Ag_2_ONP; (**b**) high-magnification TEM image of Ag_2_ONP; (**c**) high-resolution (HR)TEM image of Ag_2_ONP; (**d**) lattice spacing of Ag_2_ONP; (**e**) SAED pattern image of Ag_2_ONP.

The shape and size of Ag_2_ONPs were elucidated using TEM (Figure 4). Aliquots of Ag_2_ONPs dispersed in ethanol were placed on a carbon-coated copper grid and allowed to dry under ambient conditions, and TEM images were recorded. The TEM micrographs suggest that the sizes of the particles were around 50–60 nm. The particles were aggregated and display a prism-like shape. Additionally, Ag_2_ONP has lattice fringes with a spacing of 0.209 nm, as shown in Figure 4e, which is in agreement with the previous literature [44].

### 2.4. UV–Visible Light Spectral Analysis (UV–Vis) of Nappa Cabbage-Mediated Biosynthesis of Prism-Like Silver Oxide Nanoparticles (Ag_2_ONPs)

**Figure 5:**
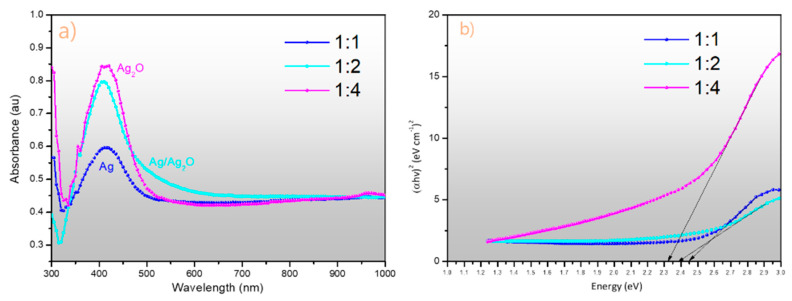
(**a**) Ultraviolet–visible light (UV–Vis) spectra of synthesized nanoparticles at different extract ratios: AgNP at 1:1 ratio, AgNP–Ag_2_ONP at 1:2 ratio, Ag_2_ONP at 1:4 ratio; (**b**) Tauc plot for band gap determination.

The UV–Vis analysis of the synthesized AgNPs and Ag_2_ONPs at different extract concentrations is shown in Figure 5a, indicating that the AgNPs synthesized at an extract-to-AgNO_3_-solution ratio of 1:1 show an absorbance band at 420 nm, whereas, for the AgNP–Ag_2_ONPs synthesized at ratio of 1:2, the bands were developed at 418 nm. In contrast, the UV–vis band observed in the Ag_2_ONPs synthesized using the extract-to-AgNO_3_-solution ratio of 1:4 was observed at 408 nm. Additionally, the Tauc plot shown in Figure 5b was investigated to see the energy band gaps of the 1:1, 1:2, and 1:4 samples, and they were found to be between 2.45 eV, 2.38 eV, and 2.33 eV respectively.

### 2.5. p-Nitrophenol Sensing Measurement

**Figure 6:**
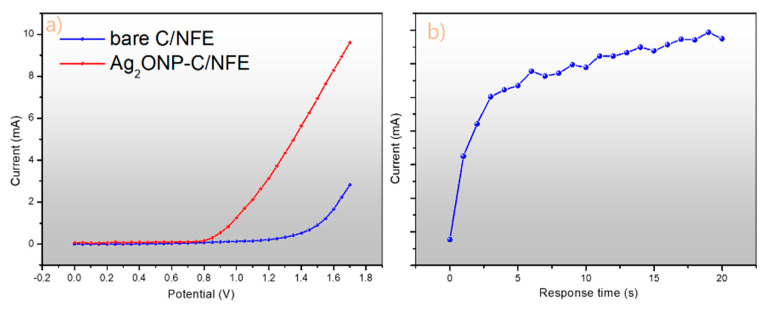
(**a**) I–V response curve of bare carbon black/nickel foam electrode and nickel foam electrode incorporating Ag_2_ONPs and carbon black (Ag_2_ONP–C/NFE) and (**b**) response time (s) at 25 μL of 0.01 mM *p*-nitrophenol (*p*-NP) added to the phosphate buffer solution pH 6.6 at 25 °C.

To test the *p*-nitrophenol sensing capability of the as-prepared Ag_2_ONPs, a nickel foam electrode was modified by incorporating Ag_2_ONPs and carbon black (Ag_2_ONP–C/NFE) where *p*-NP was detected and measured as a target analyte in the simple I–V method. The Ag_2_ONP–C/NFE fabricated electrodes have several advantages such as simplicity to construct, large surface area, good conductivity, chemical stability, bio-safe characteristics, non-toxicity, and electrochemical activity. In the case of *p*-NP sensors, the current response of Ag_2_ONP–C/NFE significantly changes when aqueous *p*-NP is adsorbed. A simple I–V method and response time experiments were performed to see the electrochemical characteristics and sensitivity of Ag_2_ONP–C/NFE toward *p*-NP. The I–V response was investigated for the bare and modified C/NFE, as shown in Figure 6a, tested at a fixed scan rate of 160 mV/s.

**Figure 7:**
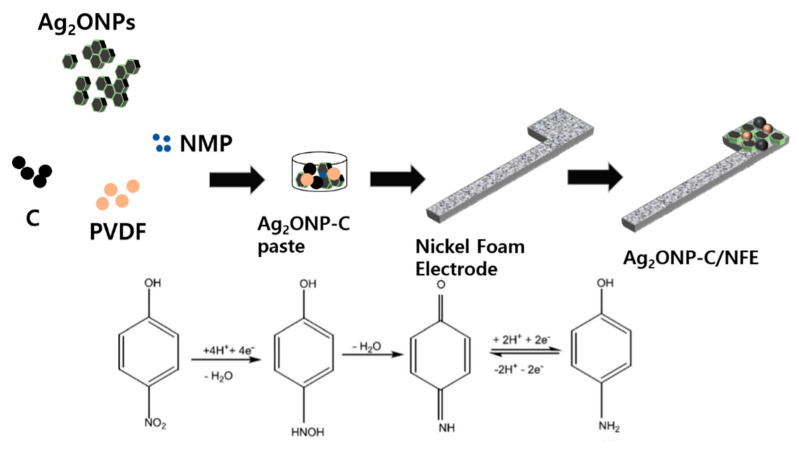
Schematic diagram of device fabrication and *p*-NP detection chemical route.

This response clearly shows the effect of modification of the carbon black/nickel foam electrode toward the *p*-NP sensitivity of the sensor. Additionally, a response time measurement of the Ag_2_ONP–C/NFE sensor was conducted as shown in Figure 6b. It was found to be around 5 s for the fabricated Ag_2_ONP–C/NFE sensor to reach the saturated current response upon addition of 25 μL of 0.01 mM *p*-nitrophenol to the electrolyte. The potential application of Ag_2_ONP–C/NFE as a chemical sensor was explored for detecting and quantifying toxic chemicals having adverse effects on the environment. The molecular structure, electron transfer mechanism, and possible reaction mechanism are generalized in Figure 7, where *p*-nitrophenol is reduced to *p*-aminophenol on the Ag_2_ONP–C/NFE sensor surface during I–V measurement.

In the presence of *p*-NP, 4e^−^/4H reduction of the nitro-group of the hydroxylamine species occurred on the Ag_2_ONP–C/NFE surface, which improved and enhanced the current responses against potential during the I–V measurement at room conditions. This suggests that the *p*-NP molecules could be adsorbed by the Ag_2_ONP–C/NFE modified electrodes and reduced. This could be due to increase in the specific surface area, adsorption sites, and conductivity of Ag_2_ONP, which increased the current response for the sensing of *p*-NP in Ag_2_ONP–C/NFE.

**Figure 8:**
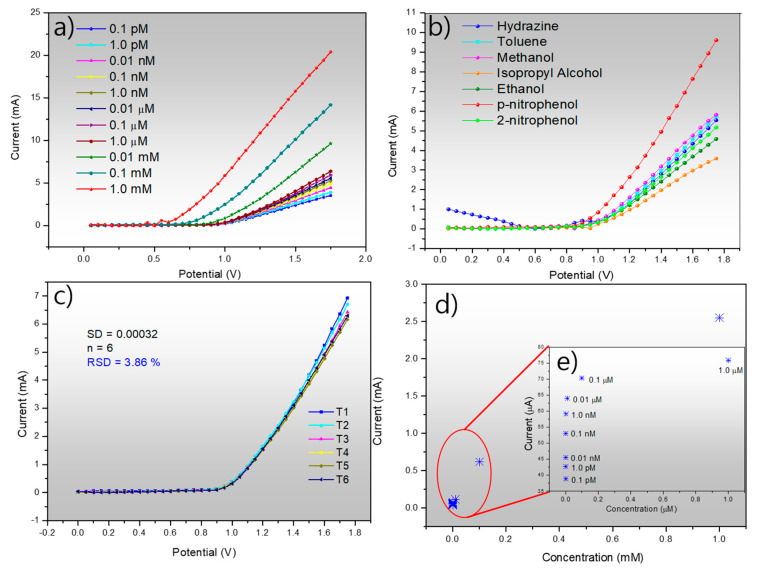
(**a**) Electrochemical detection of *p*-nitrophenol using Ag_2_ONP–C/NFE at different concentrations from 0.10 pM to 1.0 mM; (**b**) selectivity test of Ag_2_ONP–C/NFE against various analytes at 25 μL of 0.01 mM added to the phosphate buffer solution pH 6.6 at 25 °C; (**c**) reproducibility using the same working electrode; (**d**) calibration plot of Ag_2_ONP–C/NFE sensor at +0.8 V measured using 25 μL of 0.01 mM of *p*-NP; (**e**) inset: magnified view.

**Figure 9:**
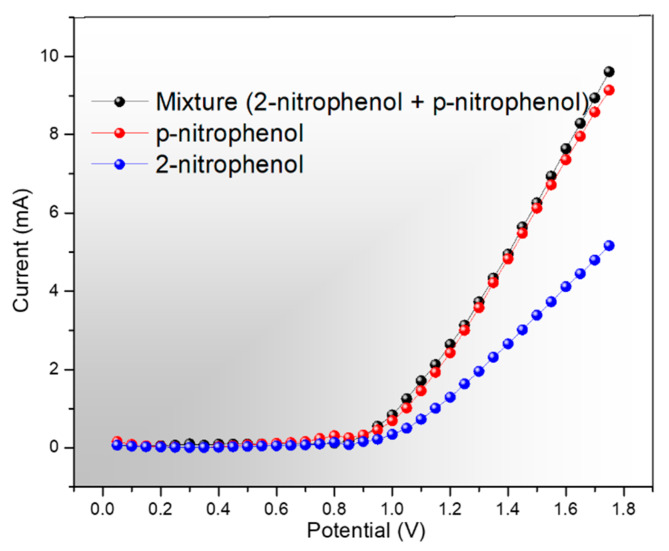
Selectivity against 2-nitrophenol (I–V response on individual analyte and mixture of 2-nitrophenol and *p*-nitrophenol.

Figure 8a shows the electrochemical response of fabricated Ag_2_ONP–C/NFE with varying concentrations of *p*-NP. The analytes were mixed with the phosphate buffer solution at pH 6.6 electrolyte at a fixed volume of 25 μL. It is clearly shown here the sensitivity of the Ag_2_ONP–C/NFE sensor for as low as a 0.1 pM concentration of *p*-NP up to 1.0 mM concentration. This could be due to the crystal structure of the as-prepared Ag_2_ONP. Additionally, it was mentioned in a previous report that the high (111) facet exposure on the morphology of crystals gives the highest conductivity compared to other index facets [45]. In accordance with this report, the Ag_2_ONP showed a high-intensity peak corresponding to the (111) plane. This characteristic of the (111) facet is due to the minimal broken chemical bond of the surface atoms of each facet—four broken chemical bonds for (100), five broken chemical bonds for (110), and finally three for (111) [46] Another, key factor in fabricating a good semiconductor device is the electron mobility that plays a very important role in sensing performance. It was previously reported that the electron holes are lighter on (111) compared to (100) and (110), thus resulting in a higher electron mobility [47]. These factors led to higher device performance of Ag_2_ONP–C/NFE sensor. Figure 8b shows the selectivity of Ag_2_ONP–C/NFE sensor toward *p*-NP against various common chemicals such as hydrazine, toluene, methanol, isopropyl alcohol, ethanol, and 2-nitrophenol. The figure clearly shows the highest current response of the Ag_2_ONP–C/NFE sensor toward *p*-NP which makes it selective to *p*-NP with respect to the chemicals tested, thus making the Ag_2_ONP–C/NFE sensor good for large manufacturing plants that use several chemicals. Along with sensor selectivity, the reproducibility shown in Figure 8c was also measured and resulted in a relative standard deviation of 3.86% upon repetition which is much lower than the previous report of 9.68% [29]. Furthermore, Figure 8d shows the calibration plot of Ag_2_ONP–C/NFE with an inset at Figure 8e to magnify the low-concentration current response at +0.8 V. Furthermore, a test against the interference of 2-nitrophenol (Figure 9) showed a comparable current response of *p*-nitrophenol in the solution and the mixture of both, which suggest an almost negligible effect of the presence of 2-nitrophenol. A comparison of proposed electrode with the previously reported modified electrode is given in Table 1. It is found that the Ag_2_ONP–C/NFE sensor showed the highest performance among the reported sensors.

## 3. Materials and Methods

### 3.1. Materials

Nappa cabbage was purchased from Greenmart located in Seoul. Silver nitrate (99.8%) and ammonium hydroxide (25.0–28.0%) were purchased from Daejung Chemicals (Siheung, South Korea). Sodium hydroxide (93.0%) was purchased from Duksan Reagents (Ansan, South Korea). Polyvinylidene difluoride, carbon black, *N*-methyl-2-pyrrolidone, hydrochloric acid, ethanol, hydrazine, toluene, methanol, isopropyl alcohol, 2-nitrophenol, phosphate buffer solution pH 6.6 at 25 °C, and *p*-nitrophenol were purchased from Sigma-Aldrich Company (St. Louis, MO, USA). All these reagents were used as received. Deionized water was used throughout the experiment.

### 3.2. Preparation of Nappa Cabbage Extract

To prepare the nappa cabbage extract, leaves were washed thoroughly with distilled water and cut into small pieces of approximately 1 cm^2^ each. Then, 10 g of these small pieces were weighed and ground in a mortar and pestle until a paste-like consistency was achieved. Next, the paste was transferred into a beaker and 100 mL of distilled water was added, followed by stirring for 2 h at room temperature. Finally, the solution was filtered two times using Whatman filter paper no. 1 and stored under 5 °C for biological synthesis of Ag_2_ONPs. To get an agreeable reproducibility, the nappa cabbage must be fresh as we want the liquid content in it for easy extraction of the phytochemicals for the reduction process.

### 3.3. Synthesis of Prism-Like Silver Oxide Nanoparticles

**Figure 10:**
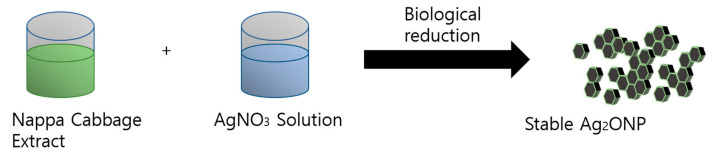
Schematic diagram of biological synthesis of Ag_2_ONP.

In this “bottom-up” approach of biological synthesis of silver oxide, the nanoparticles were formed from atoms and molecules to form the nanostructures. Figure 10 shows the schematic biological synthesis of Ag_2_ONPs.

The 0.5 mM silver nitrate solution was prepared by dissolving 1.6987 g of AgNO_3_ in 100 mL of distilled water, marked as solution A, while 1.33 g of NaOH was dissolved in 100 mL of distilled water, marked as solution B; then, the two solutions (A, B) were mixed together forming a brown solution. NH_4_OH was added to the solution drop by drop with vigorous stirring until no trace of brown precipitate was present in the solution.

For the Ag_2_ONP synthesis, different volume ratios of extract to silver nitrate solution were prepared (extract mL: silver nitrate solution mL)—40:40 mL, 20:40 mL, and 10:40 mL. The reactional mixtures were kept under magnetic stirring (500 rpm) at 130 °C for 25 min of total reaction time. The time of color change varied, resulting in the 1:1 ratio as being the fastest one to change from a pale yellow-green solution to a pale brown to black solution, indicating the formation of silver oxide nanoparticles. The nanoparticles were collected through vacuum pump filtration and washed with ethanol several times before drying at 60 °C for 12 h for 1:1, 1:2, and 1:4 samples.

### 3.4. X-ray Diffraction (XRD)

X-ray diffraction (XRD) patterns of the samples were measured using Rigaku Ultima IV with copper K_α_ (λ = 1.54059 Å) as an incident beam. The tests were carried out at room temperature with a scanning rate of 0.02°∙s^−1^ in a 2θ range of 20° to 80°. Furthermore, the grain size of the samples was analyzed by employing the Scherrer equation (Equation (1)) [57].

(1) $$\tau =\frac{k\lambda }{\beta \cos\theta },$$

where τ is the average size of the nanoparticles, k is a dimensionless shape factor (value of 0.9), λ is the wavelength of radiation, β is full width half maximum in radians, and θ is the angle diffraction.

### 3.5. Field-Emission Scanning Spectroscopy (FESEM) and Energy-Dispersive Spectroscopy (EDS)

The transition of surface morphologies of the nanoparticles was examined using field-emission scanning spectroscopy (FESEM, JEOL-7610F-Plus, Tokyo, Japan) at 15 kV. The FESEM test samples were carefully collected in glass vials. Additionally, to cover the FESEM copper plate, conductive resin tape was used where the particles were distributed and coated with gold. Element mapping was observed by energy dispersion spectrum (EDS).

### 3.6. Transmission Electron Microscopy (TEM)

The internal structure of as-synthesized Ag_2_ONP was scanned by field-emission transmission electron spectroscopy (FETEM, JEM-F200, Multipurpose Electron Microscope, Tokyo, Japan).

### 3.7. UV–Visible Light Spectral Analysis (UV–Vis)

Additionally, UV–visible light spectra of the samples were collected using a T60 UV–visible light spectrophotometer (Maharashtra, India), were scanned in the range of 300 nm to 800 nm. Finally, band gaps were computed using Tauc’s law [58].

### 3.8. Fabrication of p-Nitrophenol Sensing Electrode

As presented in Figure 7, nickel foam was used as a substrate to form the sensing device. To prepare the modified electrode, the nickel foam was firstly carefully cleaned using concentrated HCl solution (12M) in a sonicator for 5 min to remove the surface oxide layer, followed by deionized water and ethanol for another 5 min each. Next, the *p*-nitrophenol sensing electrode was prepared by creating a paste-like consistency of the following components: Ag_2_ONPs, carbon black, polyvinylidene difluoride (PVDF) with a ratio of 80:10:10, respectively, and a few drops of *n*-methyl-2-pyrrolidone. The mixture was ground in a mortar and pestle to form a homogeneous paste. Afterward, the paste was brushed onto the nickel foam electrode and oven-dried at 60 °C for 24 h.

### 3.9. p-Nitrophenol Sensing Measurement

In order to measure the *p*-NP sensing capability of the synthesized Ag_2_ONPs, the simple I–V method test was employed using Electromer, Keithley 6517A at room temperature. The electrolyte used was phosphate buffer solution at pH 6.6. Different concentrations of *p*-NP were prepared corresponding to 0.1 pM, 1.0 pM, 0.01 nM, 0.1 nM, 1.0 nM, 0.01 μM, 0.1 μM, 1.0 μM, 0.01 mM, 0.1 mM, and 1.0 mM. A uniform volume of the analyte was used throughout the experiment amounting to 25 μL mixed with 5 mL of electrolyte. The current was measured against the applied potential of fabricated silver oxide nanoparticle–carbon black/nickel foam electrode (Ag_2_ONPs–C/NFE) sensor for selective *p*-NP detection. A 0.01 mM concentration of the analyte was used in conducting the bare C/NFE and Ag_2_ONPs–C/NFE test, as well as the response time, reproducibility, and selectivity tests.

## 4. Conclusions

In summary, we synthesized silver oxide nanoparticles using nappa cabbage extract as a biological reducing agent. The extract-to-AgNO_3_-solution ratio of 1:4 favored the synthesis of Ag_2_ONPs with a morphological structure of a prism-like nanoparticle with a high intensity of the (111) facet, which is the most conductive compared to (100) and (110). This resulted in an enhanced performance of the Ag2ONPs–C/NFE toward the detection of *p*-nitrophenol. The Ag_2_ONPs–C/NFE had high sensitivity and selectivity to *p*-NP with respect to various chemicals tested at room temperature. A simple analysis procedure means that it can be applied for in-line analysis in manufacturing plants and synthesized using an eco-friendly method. These factors suggest that Ag_2_ONPs–C/NFE has wide application prospects toward *p*-NP monitoring at an ultra-low concentration range, and it could potentially be applied for *p*-nitrophenol determination in real samples.

## Figures and Tables

**Table 1:** Comparison of analytical parameters for detection of *p*-NP over various modified electrodes.

Electrode	Technique	Linear Range (μM)	Limit of Detection (μM)	Reference
β-1,4-*p*-DGA-AgNPs/GrCE	Differential pulse voltammetry	1–100	0.6	[48]
Au NPs/GCE	Semi-derivative voltammetry	10–1000	5.81	[49]
MWCNT/PDPA/GCE	Amperometry	8.9–1500	0.632	[50]
Ag NPs/GCE	Differential pulse voltammetry	0.1–350	2.57	[51]
HA-NP/GCE	Differential pulse voltammetry	1–300	0.6	[52]
Nano-Cu_2_O/GCE	Differential pulse voltammetry	1–400	0.5	[53]
Graphene/Nf/SPCE	Differential pulse voltammetry	10–620	0.6	[54]
AC/GCE	Linear sweep voltammetry	1–500	5.81	[55]
Ag particles/GCE	Differential pulse voltammetry	1–400	0.5	[56]
Ag_2_O-CNT-NCs/GCE	I–V method	0.001–10	0.000091 ± 0.000002	[29]
Ag_2_ONP–C/NFE	I–V method	0.0000001–0.01	0.0000007	This work

The abbreviations of the modified electrodes can be referred from the corresponding references.
